# Diving Behavior of the Reef Manta Ray Links Coral Reefs with Adjacent Deep Pelagic Habitats

**DOI:** 10.1371/journal.pone.0088170

**Published:** 2014-02-06

**Authors:** Camrin D. Braun, Gregory B. Skomal, Simon R. Thorrold, Michael L. Berumen

**Affiliations:** 1 Red Sea Research Center, King Abdullah University of Science and Technology Thuwal, Kingdom of Saudi Arabia; 2 Massachusetts Division of Marine Fisheries, New Bedford, Massachusetts, United States of America; 3 Biology Department, Woods Hole Oceanographic Institution, Woods Hole, Massachusetts, United States of America; Institut Pluridisciplinaire Hubert Curien, France

## Abstract

Recent successful efforts to increase protection for manta rays has highlighted the lack of basic ecological information, including vertical and horizontal movement patterns, available for these species. We deployed pop-up satellite archival transmitting tags on nine reef manta rays, *Manta alfredi*, to determine diving behaviors and vertical habitat use. Transmitted and archived data were obtained from seven tagged mantas over deployment periods of 102–188 days, including three recovered tags containing 2.6 million depth, temperature, and light level data points collected every 10 or 15 seconds. Mantas frequented the upper 10 m during daylight hours and tended to occupy deeper water throughout the night. Six of the seven individuals performed a cumulative 76 deep dives (>150 m) with one individual reaching 432 m, extending the known depth range of this coastal, reef-oriented species and confirming its role as an ecological link between epipelagic and mesopelagic habitats. Mean vertical velocities calculated from high-resolution dive data (62 dives >150 m) from three individuals suggested that mantas may use gliding behavior during travel and that this behavior may prove more efficient than continuous horizontal swimming. The behaviors in this study indicate manta rays provide a previously unknown link between the epi- and mesopelagic layers of an extremely oligotrophic marine environment and provide evidence of a third marine species that utilizes gliding to maximize movement efficiency.

## Introduction

The recent Appendix II listing by CITES of the genus *Manta* has focused attention on this enigmatic group of large pelagic rays and the human threats that have led to their vulnerable status. Both manta ray species have life history characteristics that make populations particularly vulnerable to directed and by-catch fishing [1]. Adequate conservation actions for mantas require data on horizontal and vertical movements as population connectivity plays an important role in determining the spatial scale of significant human threats [2]. While researchers had traditionally lacked the ability to track large pelagic animals *in situ*, the development of electronic tag technology has provided a wealth of information on the movements of these animals [3]. These studies have revealed a remarkable array of behaviors from ocean basin migrations [4] to individual fish dive profiles as deep as 2,000 m [5]. Yet, despite these efforts we remain remarkably ignorant of the movement patterns in many pelagic fishes, perhaps none more so than the myliobatoid (devil) rays.

The reef manta ray (*Manta alfredi*) is a small, coastal mobulid (Family: Mobulidae) that exhibits typical K-selected life history traits including slow maturation rate, small, infrequent litters, and long lifespan [1]. Sighting records suggest preferential occupation of nearshore tropical waters with strong site affinity and limited movements [6], although horizontal excursions >500 km have been documented [7]. Movements of *M. alfredi* coincide with predictable manta aggregations at several known locations in tropical and subtropical waters [1] associated with mating, cleaning, food availability, and currents [7]–[10].

Few aspects of manta biology and ecology have been adequately described, yet recent efforts have dramatically increased our knowledge about this enigmatic group [1]. Some information on horizontal movements of reef manta rays is now available [7], [8], [11], [12], but vertical habitat use remains largely undescribed and unstudied. An understanding of vertical movements is important for understanding reef manta ecology, including site occupation, feeding and mating behavior, habitat use, and potential interactions with humans. *Manta alfredi* was recently designated “Vulnerable to Extinction” by the IUCN and is listed on Appendix II of the Convention on International Trade in Endangered Species and Appendices I and II of the Convention on Migratory Species.

In the Red Sea, schools of *Manta* individuals are listed as significant features of two marine protected areas in Sudan [13], yet information on *Manta* ecology and behavior is lacking [14], [15]. Ecological information is therefore necessary for developing adequate management and conservation strategies as well as ensuring that existing protective measures are effective.

Satellite telemetry has been used to study the ecology of a diverse group of marine vertebrates [3], [16], [17]. Recent advances in this technology have enabled longer term tracking over significantly larger spatial scales than previous techniques. For instance, the use of pop-up satellite archival transmitting (PSAT) tags has facilitated the investigation of diving behavior in basking sharks by archiving high-resolution depth records for up to a year [16]. Studies on the vertical movements of *M. alfredi* have, however, been limited to active acoustic tracks, which provided high-resolution depth data over short timescales (<103 hours continuously) [11], [12], [18].

Our study provides the first use of satellite telemetry techniques to document broad-scale vertical behavior of *Manta alfredi.* Here, we seek to characterize temporal trends, habitat use, and diving in relation to manta ecology.

## Methods

**Figure 1:**
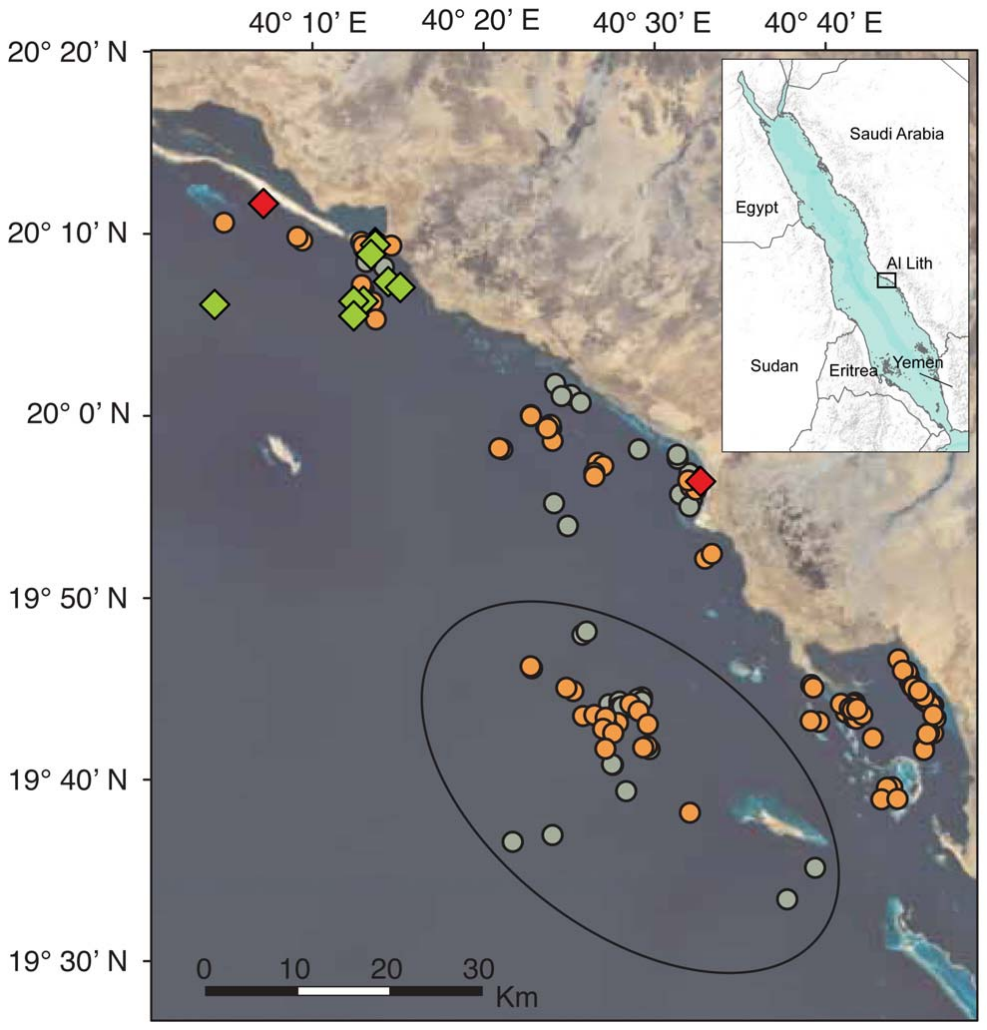
*Manta alfredi* tagging locations and habitat characterization. Locations for MA111 (orange) and MA112 (grey-green). Black oval indicates the locations characterized as “offshore” (>15 km from the coast). Green triangles indicate tagging locations for all individuals and red diamonds indicate pop-off locations for MA111 and MA112. Inset: Location of study site near Al Lith in the eastern Red Sea. Sources: Bing, U.S. Geological Survey, and ESRI.

Nine *M. alfredi* were tagged with PSAT tags off the coast of Al Lith, Saudi Arabia in the south-central Red Sea (Fig. 1) during spring 2011 and 2012 (5 miniPAT, 4 MK10-AF; Wildlife Computers Inc., USA) (Table 1). Tags were tethered to a stainless steel dart with small diameter cable and applied by a freediver with a sling spear into the dorsal musculature close to where the pectoral muscles attach to the body. This research was carried out under the general auspices of King Abdullah University of Science and Technology’s (KAUST) arrangements for marine research with the Saudi Arabian Coast Guard and the Presidency of Meteorology and Environment. The animal use protocol was performed in accordance with the Woods Hole Oceanographic Institution’s Animal Care and Use Committee (IACUC) protocol #16518 and approved by KAUST’s Biosafety and Ethics Committee.

**Table 1:** Satellite tagging summary for nine reef manta rays (*Manta alfredi*) from the Saudi Arabian Red Sea.

Manta ID	DiscWidth(cm)	Tag Type	Tag Date	Tag Lat(°N)	Tag Long (°E)	Deployment Duration (days)	PopupLatitude (°N)	PopupLongitude (°E)	Track Distance (km)
**MA102**		miniPAT	2-Apr-11	20.129	40.217	188	20.026	40.416	28
**MA103**	200	miniPAT	20-Apr-11	20.129	40.217	133	18.999	41.145	169
**MA104**		miniPAT	13-Apr-11	20.129	40.217	178	20.015	40.439	30
**MA105**	200	miniPAT	17-Apr-11	20.129	40.217	172	20.023	40.431	22.5
MA106*	250	miniPAT	16-Apr-11	20.129	40.217	155	20.166	40.200	4
MA111*	200	MK10-AF	21-Apr-11	20.154	40.229	102	20.158	40.158	15
MA112*	250	MK10-AF	22-Apr-11	20.136	40.237	102	19.928	40.549	45
**MA204**	180	MK10-AF	25-Apr-12	20.1542	40.2310	DNR			
**MA205**	200	MK10-AF	25-Apr-12	20.1558	40.2308	DNR			

Track distance refers to straight-line distance from tagging to popup locations. Two satellite tags did not report (DNR). *indicates tag was physically recovered.

Tags archived light level, depth, and water temperature every 10 and 15 seconds for the MK10-AF (MK10) and miniPAT (mP), respectively. Data were processed onboard the tag to summarize information for transmission via the Argos satellite system. Depth and temperature data were summarized every 24 (mP) and 12 (MK10) hours into 14 (mP) or 16 (MK10) bins. High-resolution time series depth data were recorded continuously (every 450 sec - mP) or cycled with one week on and two weeks off (every 75 sec - MK10) for the duration of tag deployment. At pre-programmed deployment durations of 100 (MK10) or 180 (mP) days, tags automatically popped off the animal using a corrosive burn wire. After the tags released and floated to the surface, packets of summarized data were transmitted to Argos satellites until battery failure.

Transmitted data were decoded with manufacturer software (WC-DAP 3.0, Wildlife Computers, Inc., Redmond, WA), and all subsequent analyses were conducted in the R Statistical Environment [19]. Values are expressed as mean ± SD unless stated otherwise. Dive data were stratified into day (6 am–6 pm) and night (6 pm–6 am) periods. Diel differences in depth distribution were assessed with a Two-sample Kolmogorov-Smirnov (K-S) test, and a Student’s t-test was used to compare mean day and nighttime depths. Linear least-squares regression was used to assess correlations between mean nightly depth and moon phase for each individual and a pooled dataset of all individuals. Lunar illumination data were obtained from the United States Naval Observatory (http://aa.usno.navy.mil/data/docs/MoonFraction.php, accessed Apr 2013) and arcsine-transformed before statistical analysis [20].

We recovered two MK10-AF tags that used GPS technology to acquire accurate positions of tagged animals when at the surface for sufficient time to log a position fix [21]. The GPS tracks from these two individuals were combined with high-resolution diving records to examine differences in dive behavior between coastal and offshore habitats. The two habitat characterizations were delineated by distance from shore (>15 km indicated offshore), and dive data was only considered for inclusion in either category if a GPS location was available for that day. We calculated correlations between mean nightly depth and moon fraction, vertical habitat occupation, and diel diving trends using the methods previously described.

For those tags recovered after deployment, further analysis of raw archived data included the calculation and assessment of overall dive velocities, total vertical distance travelled, and rhythmic and deep diving behaviors. Deep dives were defined as those in excess of 150 m originating or ending at depths <15 m. Ascent and descent velocities (m.s^−1^) were calculated from archived datasets (tags MA106, MA111, and MA112) as depth change over the 10 (MK10) or 15 (mP) second interval recorded by the PSAT tag. Total vertical distance traveled for each diel cycle was computed as the sum of depth changes per day and night period. Dive series were examined for rhythmic elements using autocorrelation functions in R.

## Results

Of the 9 mantas tagged, PSAT deployments ranged from 102–188 days. Four PSATs transmitted summarized data representing 5.3–54.7% of the deployment durations; two MK10-AF tags did not report, and three tags were physically retrieved. The latter tags yielded >2.6 million archived records each comprising depth, temperature, and light level records collected every 10 (MK10) or 15 (mP) seconds over the duration of tag deployment (Table 1).

**Figure 2:**
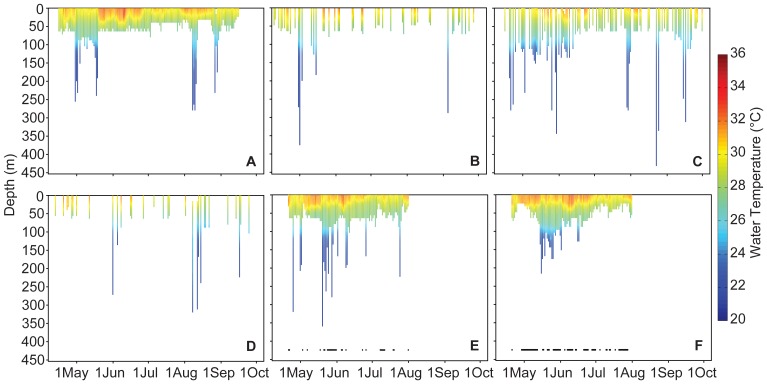
Daily ambient water temperature and depth. Daily ambient water temperature (indicated by color) and depth (y-axis) experienced by *Manta alfredi* for the duration of satellite tag deployment in 2011 near Al Lith, Saudi Arabia. (A) Manta identification number MA106 (B) MA102 (C) MA105 (D) MA104 (E) MA112 (F) MA111. Black points at the bottom of panels (E) and (F) indicate days for which the satellite tags resolved FastLoc GPS locations.

**Figure 3:**
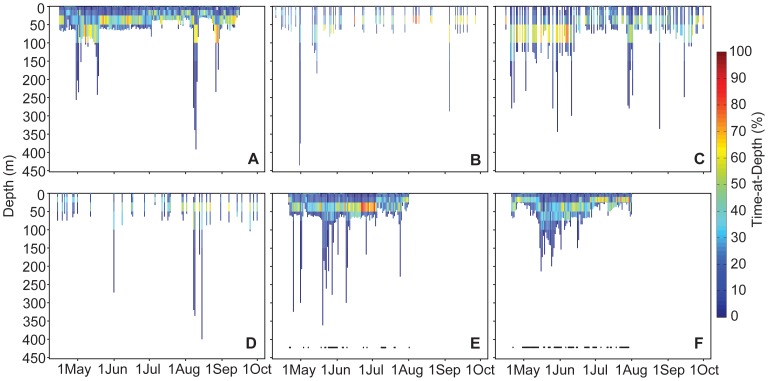
Percent daily time at depth. Daily time-at-depth (indicated by color across y-axis) by *Manta alfredi* for the duration of satellite tag deployment in 2011 near Al Lith, KSA. (A) Manta identification number MA106 (B) MA102 (C) MA105 (D) MA104 (E) MA112 (F) MA111. Black points at the bottom of panels (E) and (F) indicate days for which the satellite tags resolved FastLoc GPS locations.

Daily vertical habitat use by the tagged mantas indicated nearly constant occupation of the upper 50 m of the water column (Figs. 2,3) with sporadic use of deeper water down to 432 m (Fig. 2c). Mantas also exhibited diel vertical behavior during which the daytime depth distribution was skewed toward the upper layers and differed significantly from the nocturnal occupation of deeper waters (K-S Test p = 0.000; Fig. S1). In addition, mean daytime depths for each individual (Table 2) and all individuals combined (21.7±23.4 m) were significantly shallower than nighttime depths (34.2±21.0 m; t-test p = 0.000). Finally, regression analyses indicated that mean nightly depth was unrelated to lunar illumination for 6 of 7 individuals (Table 2, Fig. S2) and for the aggregate nightly depth data (R^2^ = 0.000, Fig. S2). Only one individual exhibited a significant but weak relationship between mean nighttime depth and lunar illumination (MA007, p<0.03, R^2^ = 0.047).

**Table 2:** Statistical results for vertical habitat use and diving behaviors of seven PSAT-tagged reef manta rays in the Saudi Arabian Red Sea.

MantaID	Day Depth (m)		Night Depth (m)			Max Velocity (m.s^−1^)	
	Mean±SE	Max	Mean±SE	Max	Lunar(R)	Descent	Ascent
MA102	24.3±0.48	341	36.0±0.37	434	0.003	–	–
MA103	23.7±0.65	59	35.0±0.61	61	0.002	–	–
MA104	23.1±0.50	363	33.9±0.37	260	0.081	–	–
MA105	35.1±0.36	312	47.6±0.30	247	0.000	–	–
MA106	21.2±0.04	392	35.3±0.03	318	0.000	1.77	1.53
MA111	20.0±0.03	214	32.7±0.03	175	0.047Ψ	2.27	1.87
MA112	23.5±0.04	362	34.7±0.03	326	0.000	1.37	1.83

All individuals exhibited a daytime depth distribution that was skewed toward upper layers compared to nocturnal depth occupation (p = 0.000). Lunar (R) values indicate the correlation coefficient for a linear regression between mean nightly depth and fraction of moon illuminated.

Ψ indicates p<0.05.

**Figure 4:**
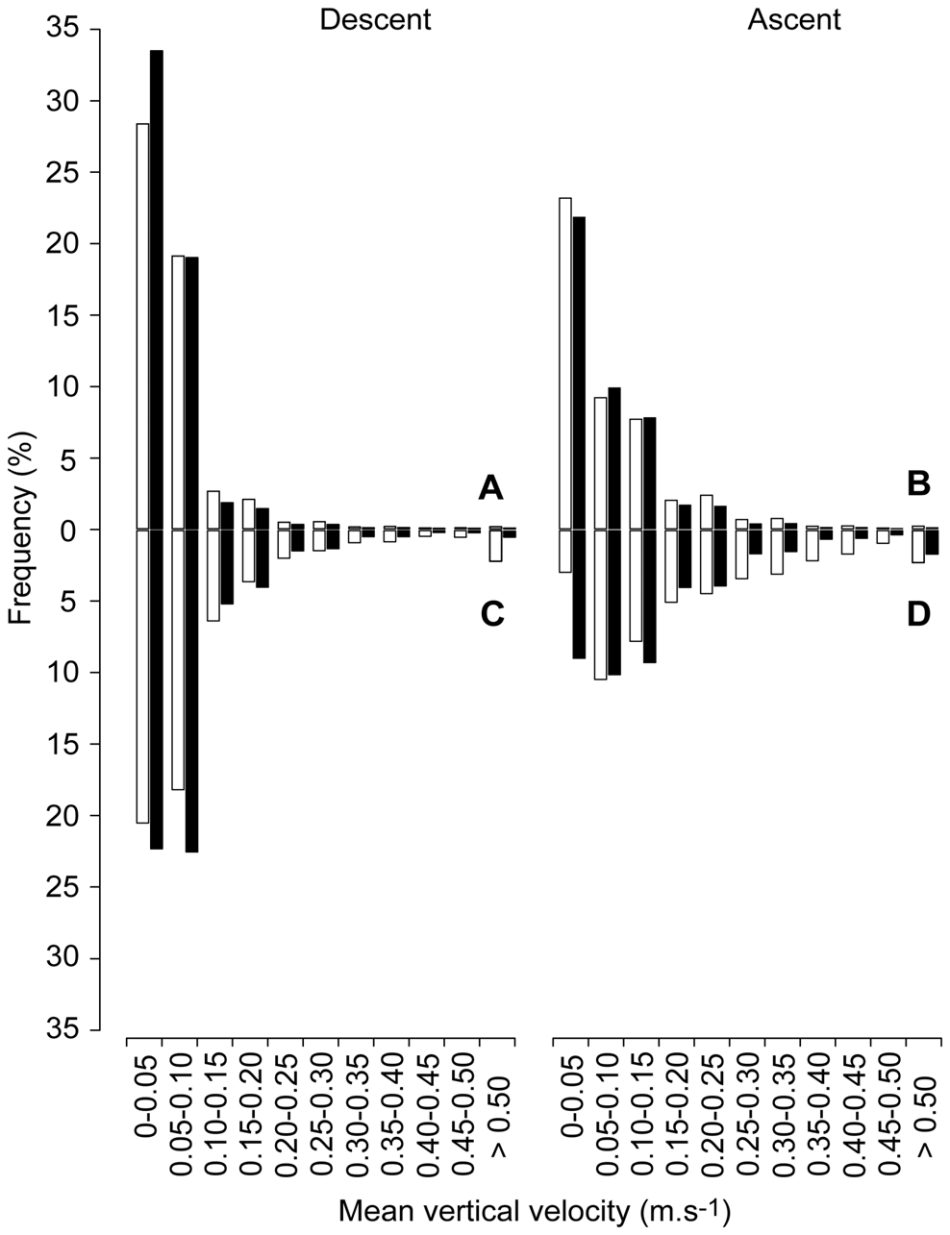
Vertical velocity during normal and deep diving. Average vertical velocity (m.s^−1^) from three archival satellite tag datasets collected during normal (<150 m) (A) descent and (B) ascent and deep (>150 m) (C) descent and (D) ascent dive behavior of *Manta alfredi*. Zero velocities (no depth change) were dropped from plotting to facilitate visualization of non-zero values. White and black bars indicate day and night, respectively.

Three recovered tags facilitated further analysis of high-resolution data sets. Vertical behaviors were dominated by slow ascents and descents (<0.25 m.s^−1^; Fig. 4a,b) with maximum rates of 1.87 and 2.27 m.s^−1^, respectively. Despite slow depth changes, these fish exhibited highly variable cumulative daily vertical movements that ranged from 2870–10,300 m. One manta (MA111) exhibited significantly more vertical movement during the day than the night (mean total daytime movement 2804±725 m and night 2528±589 m, p = 0.003) while the other two exhibited no diel differences. In addition, no rhythmic components were found using autocorrelation analyses.

**Figure 5:**
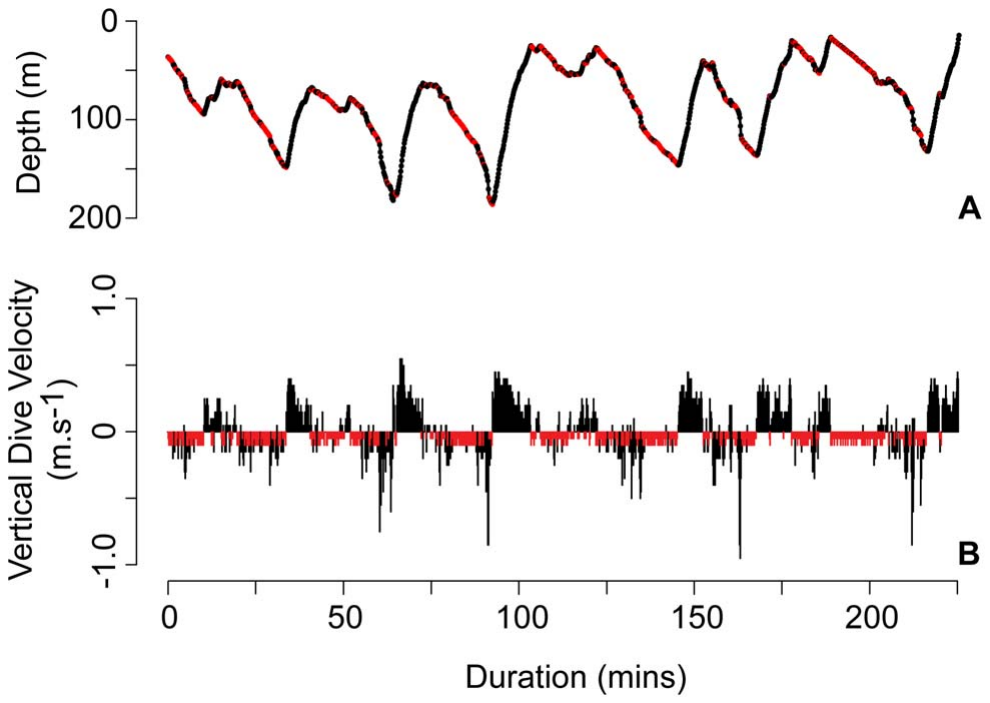
Gliding during deep dives. (A) Consecutive deep diving behavior exhibited by MA006 initiated at 0900 on 22 May 2011 and (B) the corresponding mean vertical velocity histogram. Negative velocities describe descents. Red segments indicate descent velocity between depth points <0.1 m.s^−1^ which we suggest correspond to gliding behavior.

We recorded 14 partial deep dive profiles from transmitted tag data and 62 complete deep dives (>150 m) from archival depth records that were distributed randomly throughout a daily cycle. Most descent rates derived from the cumulative deep dive record were between 0–0.10 m.s^−1^ while the ascent rate frequency distribution was skewed toward slightly faster depth changes (Fig. 4c,d). Isolation of a single series of consecutive dives highlighted the higher ascent rates compared to descent rates (Fig. 5).

**Figure 6:**
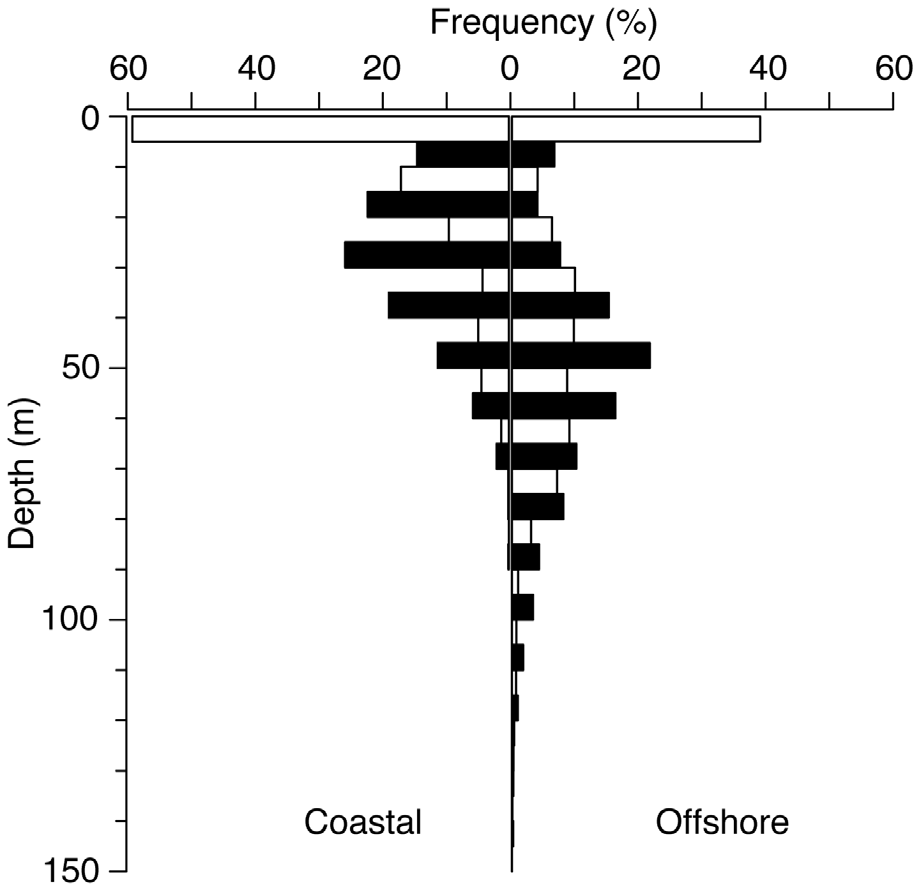
Coastal and offshore depth occupation. Frequency histogram comparing coastal (left) and offshore (right) depth occupation over a diel cycle. Bars are in 10 m bins. White and black bars indicate day and night, respectively.

**Figure 7:**
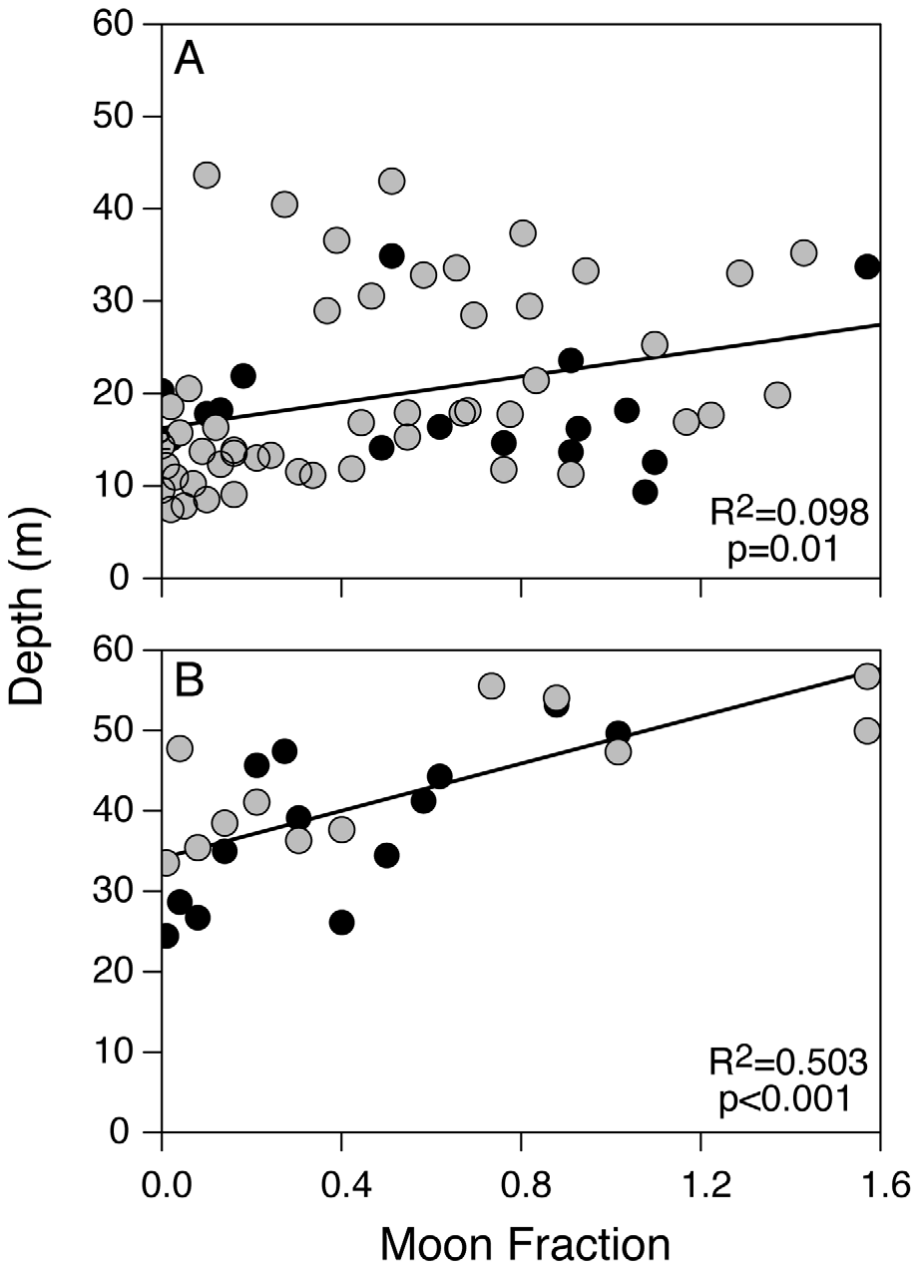
Coastal and offshore lunar regression. Moon fraction regression with mean daily nighttime depth for both GPS-tagged mantas (MA111 = grey, MA112 = black) comparing (A) coastal and (B) offshore nocturnal depth occupation.

**Figure 8:**
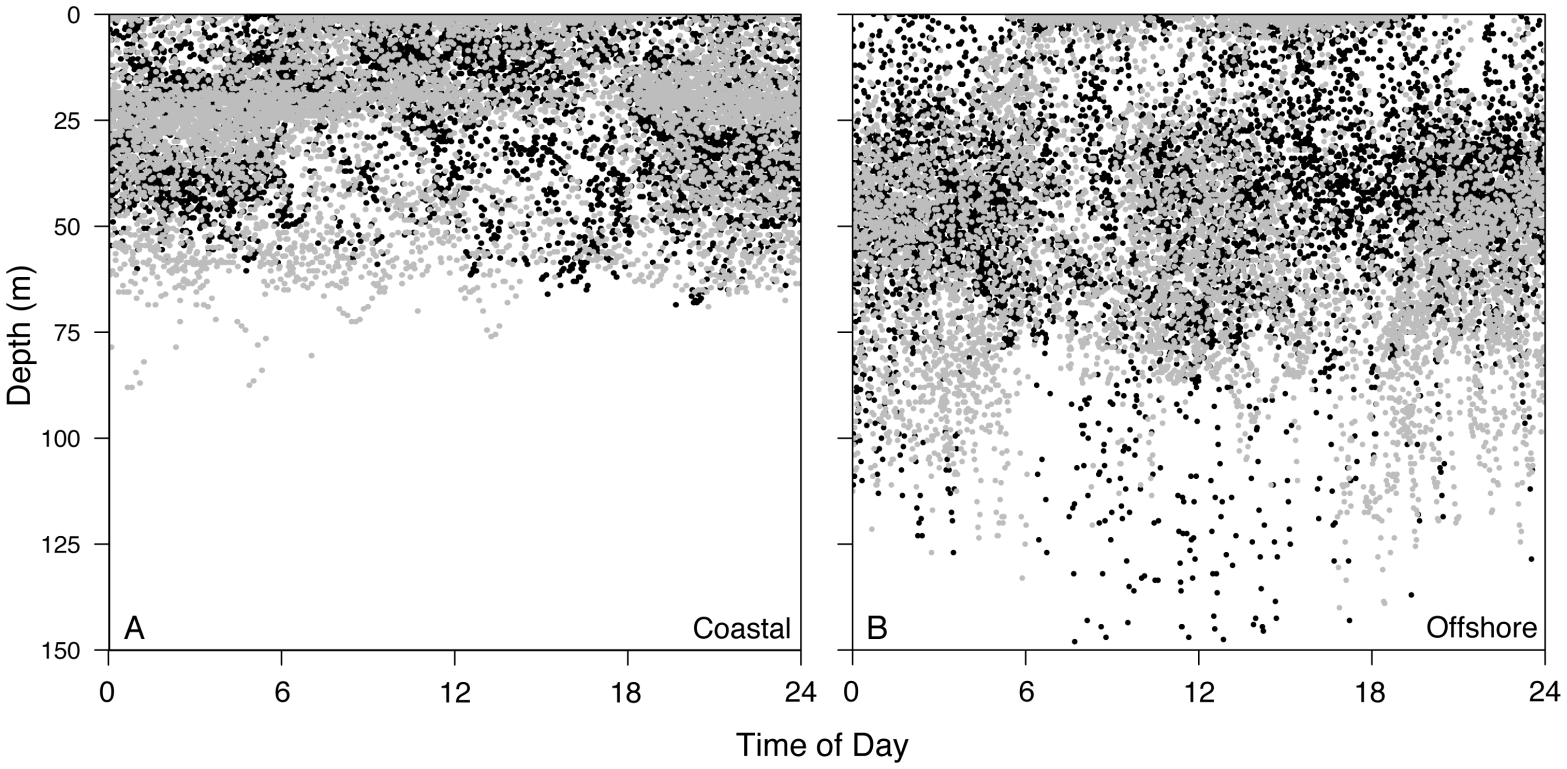
Coastal and offshore diel diving behavior. Combined diel diving behavior for MA111 (grey) and MA112 (black) during coastal (A) and offshore (B) habitat occupation. Note y-axis is truncated in (B) to facilitate comparison.

Finally, a comparison of coastal and offshore diving behavior demonstrated marked differences between habitats (Fig. 1). A total of 65 days were classified as coastal based on GPS locations (49 and 16 days for MA111 and MA112, respectively) while 25 days were considered offshore (12 and 13 days, respectively). Vertical use of the water column while in coastal habitats indicated similar depth preferences as those demonstrated when mantas were offshore but was skewed shallower, presumably as an artifact of bathymetric constraints (Fig. 6). However, regression of moon fraction (arcsine-transformed, as above) with mean nightly depth indicated a strong positive relationship between moon fraction and nighttime depth at offshore locations (R^2^ = 0.50, p<0.001), while a significant but weaker relationship was apparent in coastal environments (R^2^ = 0.098, p = 0.01, Fig. 7). Diving behavior over the diel cycle provided further contrast among coastal and offshore sites (Fig. 8). Coastal diving was indicative of reverse diel vertical migration (rDVM) in which these individuals spent much of their time at depth overnight that was punctuated by a rise to and descent from the surface at dawn and dusk, respectively. Conversely, when mantas moved offshore they exhibited a similar rDVM but with a period of deeper occupation during midday that was not evident in the coastal environment.

## Discussion

Reef mantas exhibited frequent use of the upper epipelagic layer (<60 m) of the Red Sea throughout the diel cycle. This is similar to vertical behavior reported by the few existing studies conducted on other mobulid species [11], [22], [23]. However, in contrast to other mobulids, mantas in this study demonstrated strong rDVM within the upper layer, occupying surface habitats during daytime hours and moving deeper at night. Several active and passive acoustic studies have suggested movement away from reef environments and into deeper water at night. Anderson et al. [9] speculated, based on field observations in the Maldives, that mantas regularly used a deep zooplankton layer at night. During major upwelling events, this layer appears to reach the surface where large groups of mantas were observed feeding. Similarly, manta detections on passive acoustic arrays have been shown to drastically decrease during nocturnal periods, leading the researchers to draw similar nighttime feeding conclusions [8]. In addition, Clark [11] found all actively tracked mantas made similar offshore excursions to deeper waters during the night. Until now, however, this behavior was observed in a few actively tracked animals over a relatively short duration (up to 150 hours) [11] or inferred from passive acoustics [8], [12]. Our study quantified diel depth changes over several months, and we conclude that this behavior is likely common in reef mantas throughout their range.

Reverse DVM behavior similar to that reported here has also been shown in whale sharks at Ningaloo Reef [24]. However, mantas in this study exhibited no particular affinity for surface waters specifically during dusk periods as reported for whale sharks. Rather, mantas spent most of their time at the surface during all daylight hours before descending around dusk. Previous studies have suggested that nocturnal occupation of deeper water may increase foraging opportunities in other marine species, including filter feeding elasmobranchs [25]–[28], and may be a strategy for mantas to exploit rising layers of mesopelagic zooplankton that move into the photic zone at night [29], [30] or demersal plankton emerging from the reef benthos [30], [31]. In the Red Sea, it is clear that significant plankton biomass moves shallower at night from deeper layers in the pelagic environment and emerges from the benthos in reef environments [30], [32], [33]. Both likely provide valuable food resources in an oligotrophic environment like the Red Sea. Vertical behaviors in this study suggested that mantas occupy both offshore and reef habitats in a way that facilitates exploitation of emergent reef and rising pelagic plankton. This hypothesis is supported by isotopic analysis of reef manta muscle tissue that indicated a strong demersal, reef-derived diet source [34]. Feeding on emerging reef plankton may also explain the origination of unique fatty acids found in manta tissues [35].

We were able to characterize offshore diving behavior in two mantas based on high-resolution GPS locations. These individuals occupied a single offshore area at the same time in late May 2011. During this time, both individuals exhibited midday occupation of deeper water in contrast to the rDVM shown in coastal habitats. We can only speculate as to why these rays where deeper in the water column during midday hours. Possible explanations include visits to cleaning stations [7], reproductive behavior [36], [37], or perhaps most likely the exploitation of an ephemeral food resource [9], [10]. More work will be needed to distinguish among these hypotheses.

Although light likely influences the vertical behavior of many large pelagic fishes, including billfishes [38], [39] and sharks [40], the direct effects of lunar illumination on mobulids is unclear. To date, the extent to which the lunar cycle influences manta behavior has been inferred from short-term passive acoustic studies [8], [10], [11]. Working with a more comprehensive dataset, we found that mantas overall did not alter vertical habitat use in association with the lunar cycle. However, during offshore movements, mantas in our study occupied deeper water at night as the moon phases progressed toward full. This behavior is consistent with the vertical movements of pelagic plankton as a strategy to avoid visual predation during increasing surface irradiance around the full moon [30] and indicates mantas are potentially exploiting these planktonic resources while in offshore waters. In contrast, nighttime depths of mantas exploiting coastal, reef-derived plankton as a nocturnal food source are probably unaffected by lunar illumination [30].

Mantas inhabit the full temperature range in the epi- and mesopelagic layers of the Red Sea (range 21.6–34.2°C) with dives >400 m, including a 432 m dive that extends the current depth range for *M. alfredi* by over 100 m [41]. Deep dives occurred relatively infrequently throughout all but one deployment and were characterized by asymmetrical V-shaped dive profiles with extremely short bottom times, relatively slow descents, and faster ascents. Although these dives may allow the rays to exploit the relatively rich zooplankton layers below 200 m in the Red Sea [33], this motivation seems unlikely based on the dive profiles. If the mantas were searching for food, they would presumably be successful at least occasionally, cease diving, and occupy that layer for some period of time. Yet we never observed this leveling out behavior in any of the 76 deep dives performed by 6 individuals in this study. Alternatively, these deep dives may be associated with travelling behavior in which mantas perform gliding dives to conserve energy and maximize movement efficiency. Although our tags didn’t record pitch angle and tail-beat frequency data, vertical descent velocities from the three high-resolution profiles were skewed toward slower descents than ascents. The flattened descent profile of a single series of consecutive deep dives that results from this behavior provided further visual support for the glide hypothesis and demonstrates what appeared to be a largely passive descent followed by active ascent. Interestingly, these descent rates are similar to those recorded for Japanese flounder [42]. In addition, manta ascent and descent velocities exhibited the same relationship as lateral acceleration during ascent and descent in whale sharks with very slow descent rates while consistently reaching 0.10 m.s^−1^ upon ascent [43].

Gliding in fishes involves harvesting kinetic energy from potential that is derived from the fish’s negative buoyancy upon descent. This is an efficient strategy for movement in an aqueous medium and may decrease drag threefold compared to active swimming [44]. Exceptional glide performance has been demonstrated in both Japanese flounder (*Paralichthys olivaceus*) [42] and whale sharks (*Rhincodon typus*) [43], and is a prevalent behavior among white sharks [45]. *R. typus* has large, flat pectoral fins that aid in passive descent while *P. olivaceus* exhibits significant dorso-ventral flattening capable of producing substantial lift. Mobulid rays are also dorso-ventrally flattened. Their wing-like pectoral fins can generate significant lift [46], and, therefore, they possess body types ideally suited for maximizing glide efficiency. Morphological traits for efficient movement combined with negative buoyancy make powerless glide diving followed by active ascent a more energetically efficient strategy for traversing horizontally than continuous swimming [42] and may provide significant energetic benefits to mantas during travel. Further insight and confirmation of manta gliding will require the deployment of accelerometers that can quantify three-dimensional movement at high temporal resolution [47].

Manta rays have remained largely enigmatic despite their iconic status with divers around the world. Our tag results extend the habitat of reef mantas well into the mesopelagic zone of the global ocean. Recent PSAT tagging studies have similarly extended the depth ranges of other elasmobranchs through the mesopelagic and even into bathypelagic depths [5], [16]. Prey at these depths below the epipelagic zone may provide valuable food resources in oligotrophic environments. This diving behavior may provide a new ecological connection between the surface ocean and the deep sea.

## Supporting Information

Figure S1 Diel changes in depth occupation. Distribution of percent time at depth (m) during day (white) and night (black) from aggregate depth records for nine *Manta alfredi* in the south-central Red Sea.
(TIF)

Figure S2 Lunar regression with mean nightly depth. Moon fraction regression with mean daily nighttime depth for individual reef mantas (A) MA106 (B) MA102 (C) MA103 (D) MA105 (E) MA104 (F) MA112 (G) MA111 and H) all mantas tagged with satellite tags in the Saudi Arabian Red Sea.
(TIF)
