# Supplementary figures and images for: Diving Behavior of the Reef Manta Ray Links Coral Reefs with Adjacent Deep Pelagic Habitats
Source: PLoS One. 2014 Feb 6;9(2):e88170. doi: 10.1371/journal.pone.0088170 (PMC3916408; doi:10.1371/journal.pone.0088170)

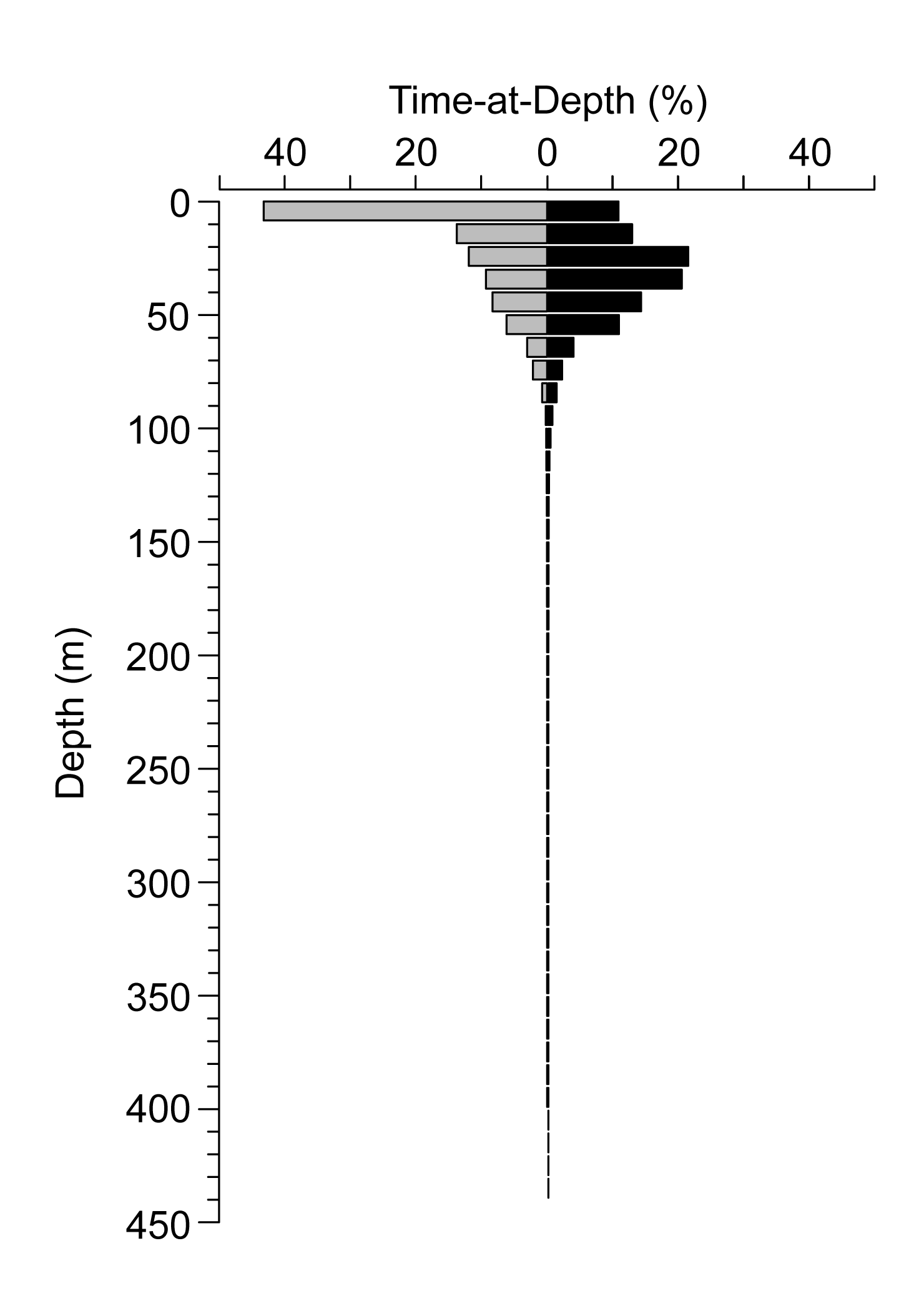

Supplement: Figure S1 — Diel changes in depth occupation. Distribution of percent time at depth (m) during day (white) and night (black) from aggregate depth records for nine Manta alfredi in the south-central Red Sea. (TIF) [file pone.0088170.s001.tif]

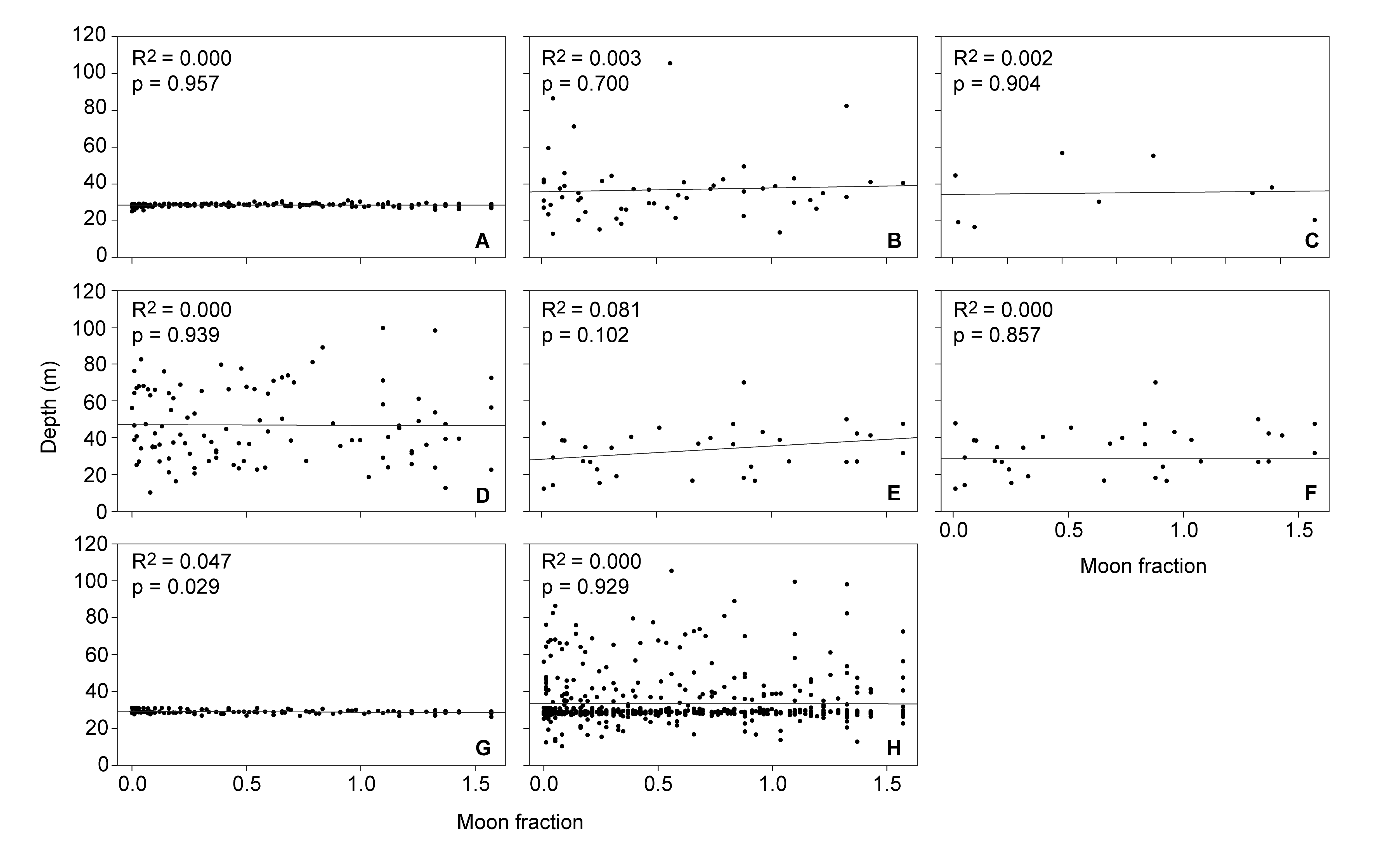

Supplement: Figure S2 — Lunar regression with mean nightly depth. Moon fraction regression with mean daily nighttime depth for individual reef mantas (A) MA106 (B) MA102 (C) MA103 (D) MA105 (E) MA104 (F) MA112 (G) MA111 and H) all mantas tagged with satellite tags in the Saudi Arabian Red Sea. (TIF) [file pone.0088170.s002.tif]
